# Guillain-Barré Syndrome: Inverted Paralysis Post-Operatively in a Patient With Ulcerative Colitis

**DOI:** 10.7759/cureus.15457

**Published:** 2021-06-05

**Authors:** Esha Jain, Deepa Nuthalapati, Harris Z Babar, Dennis Bloomfield

**Affiliations:** 1 Medicine, American University of Antigua, St. John's, ATG; 2 Neurology, International American University, St. Lucia, LCA; 3 Medicine, University of Maryland Medical Center, Baltimore, USA; 4 Cardiology, Richmond University Medical Center, Staten Island, USA

**Keywords:** guillain barre syndrome (gbs), pharyngeal-cervical-brachial (pcb) variant, ulcerative colitis (uc), gastrointestinal surgery, electromyography and electro-stimulation

## Abstract

Rapidly progressive and life-threatening, the pharyngeal-cervical-brachial (PCB) variant of Guillain-Barré syndrome (GBS) poses a unique perspective on the complications that may arise post-operatively, especially in patients with an underlying autoimmune disease like ulcerative colitis. GBS is an immune-mediated polyneuropathy typically consisting of ascending flaccid muscle paralysis and areflexia. There are several variants of GBS, which can present atypically and be misdiagnosed. We intend to focus on PCB, a rare, localized variant of GBS. Herein, we report an uncommon case of a recent post-surgical patient, with underlying ulcerative colitis, who developed the PCB variant of GBS.

## Introduction

Ulcerative colitis is a chronic and recurrent inflammatory disease, and Guillain-Barré syndrome (GBS) is a rapid-onset muscle weakness caused by the immune system damaging the peripheral nervous system. Ulcerative colitis and GBS can be caused by immune system abnormalities and can co-exist [1]. The pharyngeal-cervical-brachial (PCB) variant of GBS is defined by rapidly progressive oropharyngeal and cervicobrachial weakness associated with areflexia in the upper limbs [2]. The classic presentation of GBS is characterized by symmetrical, ascending muscle weakness usually involving the lower limbs first. Deep-tendon reflexes are absent in 90% of patients as well [3]*. *As the PCB variant is quite rare, it can often be misdiagnosed as a brain stem stroke, myasthenia gravis, or botulism [4]. The relationship of this variant to underlying autoimmune diseases and the stress of surgery is not only uncommon, but widely underreported. We intend to report the only known case of the PCB variant of GBS in a 68-year-old female with underlying ulcerative colitis following a gastrointestinal surgical procedure four weeks earlier.

## Case presentation

The patient is a 68-year-old Hispanic/Latino female with ulcerative colitis who had a prior colectomy and, more recently, a small bowel resection with ileostomy creation one month ago. She was referred from her nursing home for a one-week history of altered mental status, increasing lethargy, and worsening weakness in her extremities. The patient described the weakness beginning initially in the fingertips and upper extremities, and over the course of five days descending to her lower extremities. Simultaneously she experienced shortness of breath, low volume, and pitch of her voice, as well as difficulty swallowing. 

Physical examination on day 1 of admission showed the patient in mild distress, dyspneic, and speech was hypophonic. Neurological assessment included Glasgow Coma Scale (GCS) which was 15/15. Examination showed significant motor deficits of both upper and lower extremities. She exhibited decreased power of 2/5 and absent reflexes in the arms bilaterally. Both legs showed a power of 3/5 with absent ankle reflexes and trace reflexes present in bilateral knees. Apart from decreased soft palate motion, cranial nerve 2 - 12 examination was otherwise unremarkable. In addition, her gross sensory examination was normal. The patient’s cerebellar function and gait were unable to be assessed due to her progressing lower extremity weakness. Upon assessing the patient, the initial recommendations in accord with the primary team were to admit the patient to the ICU to optimize her hemodynamic stability, consider ventilator use (due to potential diaphragmatic paralysis), and continue monitoring her neurological symptoms. At this time, additional labs and tests were also ordered, including, negative inspiratory force (NIF) and vital capacity (VC), lumbar puncture (LP) to evaluate for albumin-cytologic dissociation, immunoglobulin A (IgA) levels, and acetylcholine-receptor (Ach-R) antibody. Results were reviewed and recorded in Table 1. Ach-R binding and modulating antibodies were not detected. This patient's NIF ranged from -26 to - 47cm of water requiring intense monitoring as a drop to -20cm H2O requires intubation. She was started on intravenous immunoglobulin (IVIG) 0.4 g/kg for 24 hours for five days which was later changed on day 4 of admission to ten days as her condition showed no improvement. Following completion of IVIG, the patient was monitored for four days for any improvement in health, and considerations were made to begin plasmapheresis. 

**Table 1:** Laboratory Results Day 1 to Day 10

Cerebrospinal Fluid (CSF) Studies:		Normal Values
Appearance	Clear	
Cell Count	0	0-5/mm^3^
Immunoglobulin G (IgG)	1.8	0-4.5mg/dL
WBC	0	<5 cells
Total Protein	30.6	15-45 mg/dL
Glucose	69	40-70mg/dL
Culture and Gram Stain	Negative	
Antibody (Ab) Studies:		
Immunoglobulin A (IgA)	325	40-700mg/dL
GQ1B	<1:100	Normal
Acetylcholine Receptor (Ach-R) Modulating	14	Normal
Acetylcholine Receptor (Ach-R) Binding	<0.30	Normal
Respiratory Studies:		
Vital Capacity (VC)	1000-1400 mL	3000-5000 mL
Negative Inspiratory Force (NIF)	-22 to -47cmH_2_O	-75 to -100 cmH_2_O

On day 7 of admission, her upper extremity weakness worsened with power of 1/5 in the left hand (ability to move thumbs), and 0/5 for right upper extremity. While her upper extremities showed no improvement in return of motor function and reflexes, we noticed very minimal return of power in her lower extremities and deep tendon reflexes in the ankles. A nerve conduction study and electromyographic (EMG) nerve test were performed and results are shown in Table 2 and Table 3. The results obtained revealed severe sensorimotor, axonal, and demyelinating peripheral neuropathy of the bilateral upper and lower extremities. All F wave latencies were below normal limits. All examined muscles showed no evidence of electrical response and muscle stretch reflexes were 0. Clinical correlation was advised for acute GBS.

**Table 2:** Motor Nerve Conduction Study of the Upper and Lower Limbs. Abnormal Values are in Bold APB, Abductor Pollicis Brevis; B Fib, Brevis Fibularis; EDB, Extensor Digitorum Brevis; AHB, Abductor Hallucis Brevis; ADM, Abductor Digiti Minimi

Nerve	Site	Onset - Peak Latency (ms)	Normal Onset - Peak Latency (ms)	Amplitude (mV)	Normal Amplitude (mV)	Site 1	Site 2
Left Median (APB)	Wrist	13.8	< 4.5	0.0	>4		
Right Median (APB)	Wrist // Elbow	13.8 // 13.8	< 4.5	0.0 // 0.0	>4 // >4	Elbow	Wrist
Left Peroneal (EDB)	Ankle // B Fib	13.8 // 13.8	< 5.8	0.0 // 0.0	>2 // >2	B Fib	Ankle
Right Peroneal (EDB)	Ankle // B Fib	13.8 // 13.8	< 5.8	0.0 // 0.1	>2 // >2	B Fib	Ankle
Left Tibial (AHB)	Ankle // Popliteal	1.1 // 13.8	< 6	1.3 // 0.0	>2 // >2	Popliteal	Ankle
Right Tibial (AHB)	Ankle // Popliteal	13.8 // 13.8	< 6	0.0 // 0.0	>2 // >2	Popliteal	Ankle
Left Ulnar (ADM)	Wrist // Elbow	13.8 // 13.8	< 3.5	0.0 // 0.0	>4 // >4	Elbow	Wrist
Right Ulnar (ADM)	Wrist // Elbow	1.3 // 13.8	< 3.5	1.3 // 0.0	>4 // >4	Elbow	Wrist

**Table 3:** Electromyography (EMG) Results Fibs, Fibrillations; PSW, Positive spike waves; Amp, Amplitude; Dur, Duration; Poly, Polyphasics; Nml, Normal

Side	Muscle	Nerve	Root	Fibs	Psw	Amp	Dur	Poly	Firing Rate
Right	Medial Gastrocnemius	Tibial	S1-2	0	0	Nml	Nml	Nml	Nml
Right	Semitendinosus	Sciatic	L5, S1-2	0	0	Nml	Nml	Nml	Nml
Right	Peroneus Longus	Superficial Peroneal	L5, S1	0	0	Nml	Nml	Nml	Nml
Right	Tensor Fasciae Latae	Superficial Gluteal	L4-5, S1	0	0	Nml	Nml	Nml	Nml
Right	Adductor Magnus	Obturator, Sciatic	L2-4	0	0	Nml	Nml	Nml	Nml
Right	Anterior Tibialis	Deep Peroneal	L4-5	0	0	Nml	Nml	Nml	Nml
Right	Vastus Medialis	Femoral	L2-4	0	0	Nml	Nml	Nml	Nml
Left	Medial Gastrocnemius	Tibial	S1-2	0	0	Nml	Nml	Nml	Nml
Left	Semitendinosus	Sciatic	L5, S1-2	0	0	Nml	Nml	Nml	Nml
Left	Peroneus Longus	Superficial Peroneal	L5, S1	0	0	Nml	Nml	Nml	Nml
Left	Tensor Fasciae Latae	Superficial Gluteal	L4-5, S1	0	0	Nml	Nml	Nml	Nml
Left	Adductor Magnus	Obturator, Sciatic	L2-4	0	0	Nml	Nml	Nml	Nml
Left	Anterior Tibialis	Deep Peroneal	L4-5	0	0	Nml	Nml	Nml	Nml
Left	Vastus Medialis	Femoral	L2-4	0	0	Nml	Nml	Nml	Nml
Right	Deltoid	Axillary	C5-6	0	0	Nml	Nml	Nml	Nml
Right	Biceps	Musculocutaneous	C5-6	0	0	Nml	Nml	Nml	Nml
Right	Triceps	Radial	C6-7-8	0	0	Nml	Nml	Nml	Nml
Right	Flexor Carpi Radials	Median	C6-7	0	0	Nml	Nml	Nml	Nml
Right	Flexor Carpi Ulnar	Ulnar	C8, T1	0	0	Nml	Nml	Nml	Nml
Right	Extensor Digitorum Communis	Radial (Posterior Interosseous)	C7-8	0	0	Nml	Nml	Nml	Nml
Right	Brachioradialis	Radial	C5-6	0	0	Nml	Nml	Nml	Nml
Right	Abductor Pollicis Brevis	Median	C8-T1	0	0	Nml	Nml	Nml	Nml
Right	1^st^ Dorsal Interosseous	Ulnar	C8-T1	0	0	Nml	Nml	Nml	Nml
Left	Deltoid	Axillary	C5-6	0	0	Nml	Nml	Nml	Nml
Left	Biceps	Musculocutaneous	C5-6	0	0	Nml	Nml	Nml	Nml
Left	Triceps	Radial	C6-7-8	0	0	Nml	Nml	Nml	Nml
Left	Flexor Carpi Radials	Median	C6-7	0	0	Nml	Nml	Nml	Nml
Left	Flexor Carpi Ulnar	Ulnar	C8, T1	0	0	Nml	Nml	Nml	Nml
Left	Extensor Digitorum Communis	Radial (Posterior Interosseous)	C7-8	0	0	Nml	Nml	Nml	Nml
Left	Brachioradialis	Radial	C5-6	0	0	Nml	Nml	Nml	Nml
Left	Abductor Pollicis Brevis	Median	C8-T1	0	0	Nml	Nml	Nml	Nml
Left	1^st^ Dorsal Interosseous	Ulnar	C8-T1	0	0	Nml	Nml	Nml	Nml

Over the next week, we were able to see a remarkable improvement in motor function, specifically more return of function in the legs versus the arms. The patient was able to move both her feet side to side as well as exhibit successful abduction and adduction of the fingers bilaterally. On day 25, she started ten sessions of plasmapheresis and was recommended for neuromuscular clinic evaluation along with acute rehabilitation after discharge. Recommendation was also placed to continue IVIG treatment outpatient. On day 35, she was cleared for discharge. At this time, muscle tone was decreased more in the upper extremities versus the lower extremities. Power of bilateral lower extremities showed improvement at 2/5, with power of bilateral upper extremities remaining at 1/5.

## Discussion

GBS is an acute immune-mediated inflammatory polyneuropathy typically characterized by progressive ascending lower extremity weakness, usually following an antecedent trigger [5]. The most common infectious agents include Campylobacter jejuni, cytomegalovirus (CMV), and Mycoplasma pneumonia. Surgery, trauma, and immunizations have also been associated with the onset of GBS [6]. 

The PCB variant of GBS can be characterized by facial palsy, dysarthria, upper extremity muscle weakness, and areflexia of the upper limbs. The power of the lower limbs is usually preserved or only mildly affected, which occurs in the acute inflammatory demyelinating poly-radiculopathy type (the most common form of GBS) [7]. The cardinal symptoms displayed by our patient included rapidly progressing weakness of the oropharyngeal, neck, and upper extremity muscles, and areflexia, resulting in weakness, dysphagia, and hypophonia. Unlike the typical presentation of the PCB variant (which tends to spare the lower extremities), our patient displayed progression of her muscle weakness to her lower extremities, four weeks after undergoing small bowel resection due to ulcerative colitis. Being cognizant of this rapidly progressing illness, we placed our patient in the intensive care unit pre-emptively in case she may suffer diaphragmatic paralysis and prophylactically started IVIG. By prompt recognition of the patient's clinical presentation, along with confirmatory findings on diagnostic EMG, we were able to confirm the diagnosis and provide successful treatment in a timely manner. 

The patient’s mainstay of treatment primarily consisted of immunoglobulin and plasmapheresis. We observed both treatments immunoglobulin and plasmapheresis to be equally effective. While immunoglobulin therapy can block the damaging antibodies, plasmapheresis can remove the antibodies that contribute to the immune system's attack on the peripheral nerves. While there was no change in her initial medication regime, the patient was also given supplemental oxygen via nasal cannula to assist with her breathing [5].

Multiple studies have shown the development of GBS following surgeries and linked them to patients with underlying ulcerative colitis. One study found that malignancy and comorbid autoimmune disease were strongly and independently associated with the development of post-surgical GBS. Patients with post-surgical GBS were more likely to have comorbid autoimmune conditions compared to those who developed GBS without a prior surgery [8]. While surgery and acute infections have been independently linked with GBS, there are no known cases associating the development PCB variant in patients with chronic inflammatory states undergoing a surgical procedure.

## Conclusions

This patient exemplified how the PCB variant of GBS can present in an atypical fashion. Clinicians can gain to understand the many presentations of this variant as it can often be misdiagnosed. The purpose of this case report is to shed awareness on this variant as a potential complication that may arise in patients with autoimmune diseases following surgical procedures. We believe that this case study can shed light on possible rare, yet life-threatening complications that may arise post-operatively. 
